# Development of a Real-Time Fluorescence Loop-Mediated Isothermal Amplification Assay for Rapid and Quantitative Detection of *Fusarium oxysporum* f. sp. *cubense* Tropical Race 4 In Soil

**DOI:** 10.1371/journal.pone.0082841

**Published:** 2013-12-20

**Authors:** Xin Zhang, He Zhang, Jinji Pu, Yanxiang Qi, Qunfang Yu, Yixian Xie, Jun Peng

**Affiliations:** 1 Ministry of Agriculture Key Laboratory of Integrated Pest Management on Tropical Crops, Environmental and Plant Protection Institute, Chinese Academy of Tropical Agricultural Sciences, Haikou, China; 2 State Key Laboratory of Agro-biotechnology and Ministry of Agriculture Key Laboratory for Plant Pathology, China Agricultural University, Beijing, China; Nanjing Agricultural University, China

## Abstract

*Fusarium oxysporum* f. sp. *cubense* (Foc), the causal agent of Fusarium wilt (Panama disease), is one of the most devastating diseases of banana (*Musa* spp.). The Foc tropical race 4 (TR4) is currently known as a major concern in global banana production. No effective resistance is known in *Musa* to Foc, and no effective measures for controlling Foc once banana plants have been infected in place. Early and accurate detection of Foc TR4 is essential to protect banana industry and guide banana planting. A real-time fluorescence loop-mediated isothermal amplification assay (RealAmp) was developed for the rapid and quantitative detection of Foc TR4 in soil. The detection limit of the RealAmp assay was approximately 0.4 pg/µl plasmid DNA when mixed with extracted soil DNA or 10^3^ spores/g of artificial infested soil, and no cross-reaction with other relative pathogens were observed. The RealAmp assay for quantifying genomic DNA of TR4 was confirmed by testing both artificially and naturally infested samples. Quantification of the soil-borne pathogen DNA of Foc TR4 in naturally infested samples was no significant difference compared to classic real-time PCR (*P*>0.05). Additionally, RealAmp assay was visual with an improved closed-tube visual detection system by adding SYBR Green I fluorescent dye to the inside of the lid prior to amplification, which avoided the inhibitory effects of the stain on DNA amplification and makes the assay more convenient in the field and could thus become a simple, rapid and effective technique that has potential as an alternative tool for the detection and monitoring of Foc TR4 in field, which would be a routine DNA-based testing service for the soil-borne pathogen in South China.

## Introduction

Fusarium wilt of banana (Panama disease), caused by the soil-borne fungus *Fusarium oxysporum* f. sp. *cubense* (Foc), is a serious disease in banana (*Musa* spp.) production worldwide [1]–[3]. Foc can affect species of *Musa* and *Heliconia*, and strains have been classified into four physiological races. Race1is pathogenic to ‘Gros Michel’(AAA) and ‘Silk’ (AAB) [4]; Race 2 affects only the hybrid triploid Bluggoe (ABB) [5], and race 4 attacks Cavendish cultivars and all cultivars susceptible to races 1 and 2, which is considered the most important because it affects cultivars that produce more than 80% of the world’s bananas [3], [6]. The race 4 isolates were subdivided into subtropical race 4 (ST4) and tropical race 4 (TR4). The ST4 isolates cause disease in Cavendish bananas in the subtropics [6]–[8], and TR4 isolates are pathogenic both under tropical and subtropical conditions [9]–[12]. In South China, Fusarium wilt of Xiang Jiao (AAA, Cavendish bananas) was first reported in Guangdong Province in 2001 [13], which caused by TR4 [14].

To the best of our knowledge, there are currently no fungicides available to control Fusarium wilt of banana once plants were infected. Chemical control is difficult because the chlamydospores can survive in the soil. The best option is planting resistant cultivars, such as Fusarium wilt-resistant bananas selected via genetic variability from tissue [15], and transgenic bananas [16], [17]. However, Fusarium wilt of banana is still a major threat to banana production worldwide. Quarantine policies and Foc-free tissue culture planting materials are the important approaches to prevent disease spreading [18]. Therefore, it is necessary to develop an efficient and reliable technique to detect Foc in soil before bananas are planted.

The real-time PCR is applied widely for a number of soil-borne plant pathogens, such as *Helminthosporium solani* [19], *Colletotrichum coccodes* [20], [21], *Pythium* spp. [22], *Polymyxa graminis* [23], *Rhizoctonia solani* [24], *Verticillium dahliae* [25], *Phytophthora infestans* [26], *Plasmodiophora brassicae* [27], *Fusarium oxysporum* f. sp. *lycopersici* and differential determination of its races [28].

Apart from classic real-time PCR widely used in quantitative detection of soil-borne pathogens, an alternative technique termed loop-mediated isothermal amplification (LAMP) is also available used for quantitative analysis. The LAMP assay is performed under isothermal conditions and employs a DNA polymerase with strand-displacement activity. A set of four specially designed primers, which recognize a total of six distinct sequences on the target DNA, are used to amplify the product. The amplicons contain single-stranded loops, allowing primers to bind without the need for repeated cycles of thermal denaturation [29], [30]. As the LAMP reaction progresses, the by-product pyrophosphate ion forms a white precipitate of magnesium pyrophosphate. The increase in the turbidity due to the production of white precipitate correlates with the amount of DNA synthesized. Various other detection formats can be used as well. Positive LAMP reaction can be visualized with the naked eye by adding DNA-intercalating dyes such as ethidium bromide, SYBR Green I, propidium iodide and Quant-iT PicoGreen, or metal-ion indicators such as hydroxynaphthol blue (HNB) [31], CuSO_4_ [32] and calcein [33]. The generation of LAMP products can also be monitored in real-time by measuring the increase in turbidity deriving from magnesium pyrophosphate formation to infer increases in amplified DNA concentration, allowing quantitative detection of the target [34]–[37]. Lately, the detection of amplified products through fluorescence dye with an ESE-Quant tube scanner was developed. The method doesn’t need expensive equipment or reagents, and is a more simple and cost-effective technology, compared to other DNA-based tests [38], [39].

Several PCR-based assays were developed to detect Foc race 4 *in planta* and are distinguishable for Foc race 4 from non-Foc race 4 isolates [40]–[42]. Recently, real-time PCR [43] and loop-mediated isothermal amplification assay (LAMP) have been developed to detect the Foc race 4 in banana tissues [44]. Although these established methods could detect the Foc isolates in plant tissues, Foc is a soil-borne pathogen that can survive in soil and is hardly to control once banana plants were infected.

Thus, with the aim of developing effective control strategies, accurate and reliable methods for detecting and quantifying the pathogens in soil are required. The objectives of this study were to develop a Foc TR4 specific RealAmp assay, and to use this assay as a quantitative measure for direct detection of Foc TR4 in naturally infested soil samples. The feasibility of the LAMP-based quantitative detection assay was verified by testing both artificially and naturally infested soil samples in comparison to classic real-time PCR method. Additionally, the RealAmp products were also detected directly by visual observation with an improved closed-tube detection system by adding the SYBR Green I fluorescent dye to the inside of the lid prior to amplification, which is a more convenient diagnostics in filed surveys.

## Materials and Methods

No specific permits were required for the described field studies in the manuscript. The fields are located in suburb of urban districts in South China, which are used for banana growing. The locations are not privately-owned or protected in any way. The field study is about banana and its one of pathogens *Fusarium oxysporum* f. sp. *cubense*. They are not endangered or protected species.

### Source of Isolates

*F. oxysporum* isolates, and other isolates used in this study are listed in Table 1. A single spore culture of each tested isolate was grown on a Nash-PCNB plate (1.5% peptone, 2% agar, 0.1% KH_2_PO_4_, 0.05% MgSO_4_^.^7H_2_O, 0.1% pentachloronitrobenzene, 0.03% streptomycin and 0.1% neomycin). The isolates were maintained in a collection at the Institute of Environmental and Plant Protection, Chinese Academy of Tropical Agricultural Sciences, PR China.

**Table 1:** Fungal species and isolates used to test the specificity of the RealAmp assay.

Species/isolates	Original hosts	Origin
*Fusarium oxysporum* f. sp. *cubense*race 1	Banana (*Musa* spp.)	ACCC*31277
*F. oxysporum* f. sp. *cubense* ST4	Banana	ACCC31276
*F. oxysporum* f. sp. *cubense* TR4	Banana	ACCC31272
*F. oxysporum* f. sp. *cubense*	Banana	ACCC31273
*Curvularia lunata*	Banana	ACCC36365
*Colletotrichum gloeoaporioids*	Banana	ACCC31244
*Alternaria alternata*	Banana	ACCC37607
*Cordana musae*	Banana	ACCC36965
*Corynespora cassiicola*	Rubber tree	CFCC^+^82971
*Mycosphaerella fijiensis*	Banana	ACCC31400
*F. oxysporum* f. sp. *cubense*	Banana	ACCC31271
*Mycosphaerella fijiensis*	Banana	ACCC31409
*F. oxysporum* f.sp. *niveum*	Watermelon	ACCC30024
*F. oxysporum* f. sp. *cubense* race 1	Banana	ACCC31275
*F. oxysporum* f. sp. *cucumerium*	Cucumber	ACCC30220
*F. oxysporum* f. sp. *vasinfectum*	Cotton	ACCC30223
*F. oxysporum* f. sp. *pisi*	Pea	ACCC31037
*Fusarium oxysporum* f.sp.*vasinfectum*	Cotton	ACCC 36879
*Fusarium oxysporum* f.sp.*vasinfectum*	Cotton	ACCC 36887
*Fusarium oxysporum*	Vanilla	ACCC31306
*F. oxysporum* f.sp. *niveum*	Watermelon	ACCC 36173
*Fusarium solani*	Smoke tree	CFCC82471
*Verticillium dahliae*	Cotton	ACCC36204

* ACCC is Agricultural Culture Collection of China; ^+^CFCC is China Forestry Culture Collection Center.

### Mycelium and Microconidia Preparation

For genomic DNA extraction, 10 pieces of agar culture (ca. 1×1×2 mm) obtained from the margin of 3-day-old colonies growing on potato dextrose agar (PDA) plates were placed in 100 ml of potato dextrose broth (PDB, 20 g glucose and 200 g potato extract in 1 L H_2_O) medium in a 250-ml flask. After incubation in the dark at 25°C on a shaker for 6 days, the mycelia were collected on filter paper and stored at –70°C until use. Microconidia of the fungus Foc TR4 were prepared by growing plate cultures on PDA at 25°C for 10 days in darkness. Microconidia were harvested from the plates by rubbing the surface mycelium gently with a rubber swab and collecting the spores in distilled water. Hyphal debris was removed from the spores by centrifuging the crude spore preparation through a 40% sucrose pad, with the spores settling to the bottom of the tube and the rest of the cellular debris remaining on the surface of the sucrose pad. Spores were adjusted to the desired concentration (10^8^ spores/ml) by counting them in a hemocytometer for soil inoculation.

### Preparation of Soil Samples

For the artificially infected soil samples, 1-ml titers of the Foc TR4 spore suspension (about 10^8^ spores) were inoculated onto 10 g of twice-autoclaved soil substrate in 15-ml conical tubes and cultured at 25°C for 10 days. Samples were then air-dried at ambient temperature (about 3 days) and subsequently ground in liquid nitrogen to produce a fine powder, which was stored at −70°C prior to DNA extraction. DNA from the equal un-inoculated Foc TR4 spores suspension (about 10^8^ spores) for parallel test was extracted according to the manufacturer’s instructions using E–Z 96® Fungal DNA Kit (Omega, USA).

Samples of naturally infested soil were collected from two different types of banana production areas from South China in 2010–2011, including Hainan Island, Guangdong Province, Guangxi Province and Yunnan Province, respectively (Table S1). One type is the area where banana was previously planted. 15–20 soil samples were randomly collected taken from each chosen field. The other type is the banana-growing area with typical Fusarium wilt appearance. The samples were directly taken under the infected banana root. All of the soil samples were taken from within 100 mm depth and 10 mm in diameter according to Ophel-Keller *et al*. (2008) [45]. Samples were transported to a storage building and stored for a maximum of 2 weeks at a temperature of approximately 25°C. A total of 136 soil samples were collected and monitored with a minor modification soil genomic DNA extraction method described as followed.

### Extraction of Genomic DNA

DNA extraction from cultures was done using E-Z 96® Fungal DNA Kit (Omega, USA), according to the manufacturer’s instructions. Approximately 100 mg of freeze-dried mycelium or conidia were ground in liquid nitrogen for total genomic DNA isolation.

DNA extracted from soil samples according to Li and Hartman. (2003) [46] and Brierley *et al*. (2009) [21]. Briefly, 10 g air-dried soil sample was lyophilized with liquid nitrogen and then suspended in 20 ml SPCB extraction buffer (120 mm sodium phosphate, 2% hexadecyltrimethylammonium bromide (CTAB), 1.5 M NaCl, 1% polyvinylpolypyrrolidone (PVPP), 2% β-mercaptoethanol, pH 8.0) [19], [21]. After vortexing for 1 min, samples were incubated at 65°C for 30–60 min. The samples were subsequently centrifuged at 12,000 *g* for 10 min to remove soil and debris and 10 ml of 20% (w/v) sodium dodecyl sulfate (SDS) was added to the supernatant to make a final concentration of 1%. The samples were incubated at 65°C for a further 30 min and centrifugated at 12,000 *g* for 10 min, the supernatant was extracted with an equal volume of chloroform, mixed and re-centrifuged (12,000 *g* for 10 min). DNA in the aqueous phase was precipitated with 0.3 M sodium acetate (pH5.2) and an equal volume of isopropanol at −20°C for at least 2 h or overnight. DNA was pelleted by centrifugation (12, 000 *g* for 10 min), washed in 75% ethanol, and dissolved in 100 µl TE (10 mM Tris-HCl, 1 mM EDTA, pH 8.0). The extracted DNA was purified followed by a combined spin column with both PVPP and Sephadex G-75 described in Cullen *et al*. (2001) [19]. Purified DNA was collected and then mixed in a new sterile 1.5 ml tube.

All DNA samples were eluted with 100 µl Tris-EDTA (TE) buffer and stored at −70°C until required. The DNA concentration was determined using a NanoDrop spectrophotometer ND-2000 (Thermofisher Scientific, Loughborough, UK).

### LAMP Primer Design

**Figure 1:**
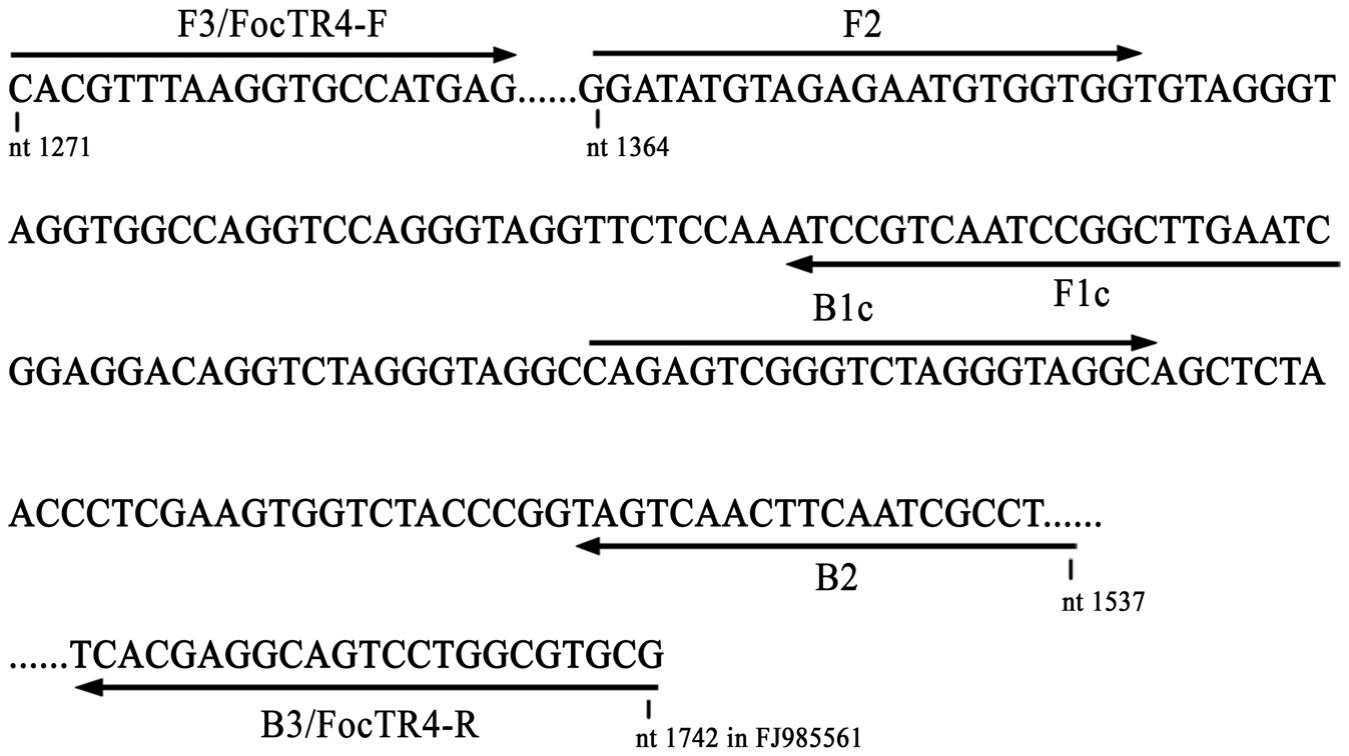
Nucleic acid sequence of intergenic spacer (IGS) region of the nuclear ribosomal operon (GenBank accession number FJ985561) used for designing inner and outer primers. The specific sequences used for primer design and their relative positions in the genome are indicated by arrows.

LAMP primers were designed according to the sequence of intergenic spacer (IGS) region of the nuclear ribosomal operon (accession number FJ985561) using PrimerExplorer V4 software (Eiken Chemical Co. Ltd., Tokyo, Japan) (http://primerexplorer.jp/e/). A forward inner primer FIP (5′- *ATTCAAGCCGGATTGACGGATT-*GGATATGTAGAGAATGTGGTGG-3′) consisted of F1c (the complementary sequence of F1, 5′-*ATTCAAGCCGGATTGACGGATT*-3′, nt 1444-1423) and F2 (5′- GGATATGTAGAGAATGTGGTGG-3′, nt 1364–1385), and a backward inner primer BIP (5′- CCAGAGTCGGGTCTAGGGTAG-*AGGCGATTGAAGTTGACTAC*-3′) consisted of B1c (the complementary sequence of B1, 5′-CCAGAGTCGGGTCTAGGGTAG-3′, nt 1467–1487) and B2 (5′- *AGGCGATTGAAGTTGACTAC*-3′, nt 1537-1518). The Foc TR4 specific primer set FocTR4-F/FocTR4-R (5′-CACGTTTAAGGTGCCATGAGAG-3′, nt 1271–1290; 5′- *CGCACGCCAGGACTGCCTCGTGA* -3′, nt 1742-1720) served as outer primer and used for the initiation of LAMP reaction [41]. All the primers were purified by HPLC (Sangon Biotec, Shanghai, China). The primer sequences and their respective binding sites were indicated in Fig. 1. The primer specificity was checked using the basic local alignment search tool (BLAST) against human DNA and other fungi sequences in the nonredundant GenBank database. Additionally, the sequences from various *formae specials* of *Fusarium oxysporum* were examined to identify the specificity of nuclear ribosomal operon regions in Foc TR4 genome.

### RealAmp Assay

The LAMP reaction contained 1.6 µM each of FIP and BIP, 0.2 µM each of F3 and B3, 12.5 µl LAMP reaction buffer containing 1.6 mM dNTPs, 1 M betaine, 8 mM MgSO_4_, 20 mM Tris-HCl (pH 8.8), 10 mM KCl, 10 mM (NH_4_)_2_SO_4_ and 0.1% Triton X-20 (Deao Biotechnology Co., Ltd, Guangzhou, China), 8 U of *Bst* DNA polymerase (New England Biolabs, Ipswich, MA), 0.2 µM SYTO-9 fluorescent dye (Invitrogen, Carlsbad, CA), 1 µL of template DNA, and double-distilled water to a final volume up to 25 µL. Then, an equal volume of paraffin oil was added to the tube to prevent evaporation, followed by adding 1 µL of 1∶10 dilution SYBR Green I (Invitrogen, Carlsbad, CA) to the inside of the lid prior to amplification with an improved close-tube visual detection system. The RealAmp assay was carried out at 63°C for 90 min using the ESE-Quant Tube Scanner (ESE Gmbh, Stockach, Germany), which was set to collect fluorescence signals at 1 min intervals. The ESE-Quant Tube Scanner is a small easy-to-use fluorescence measurement system which has an eight tube holder heating block with adjustable temperature settings and spectral devices to detect amplified product with fluorescent dye [39].

The threshold validation test is used to identify that the signal has increased sufficiently to be deemed positive. During the real-time amplification, the fluorescence data were obtained on the 6-carboxyfluorescein (FAM) channel (excitation at 487 nm and detection at 525 nm), and a fluorescence units threshold value was used, and threshold time (*Tt*) calculated as the time at which the fluorescence equaled the threshold value. The threshold value is 10 times standard deviation of the fluorescence signal during initial 5 minutes. In the plot, the Y-axis denotes the fluorescence units in milli-volts (mV) and the X-axis shows the time in minutes.

After the reaction, the LAMP products were detected directly by visual observation of the solution colour by mixing the pre-added 1 µl of SYBR Green I to the reaction solution through gentle centrifugation. Green fluorescence was clearly observed with the naked eye in the positive reaction, whereas the colour remained the original orange in the negative reaction. The RealAmp products (5 µl) were analyzed by electrophoresis on a 2% (w/v) agarose gel and subsequently stained with ethidium bromide.

### Specificity and Sensitivity Test of RealAmp Assay

To confirm the specificity of the RealAmp assay, the DNAs of other relative fungi, *Mycosphaerella melonis*, *Fusarium oxysporum* f. sp. *cucumerium*, *Fusarium oxysporum* f. sp. *lactucae, Fusarium oxysporum* f. sp. *luffae, Fusarium oxysporum* f. sp. *cubense* race 1 (Foc1) and subtropical race 4 (ST4) were used in the analyzes. In addition, the smallest fragment from RealAmp amplification products were cloned and sequenced. The specificity was also validated by conventional PCR using the specific primer set FocTR4-F/FocTR4-R previously reported by Dita *et al*. (2010) [42], which produced a 463-bp specific amplified fragment.

To determine the sensitivity of the RealAmp assay, a 463-bp-specific DNA fragment containing the LAMP target region was amplified by PCR using the specific FocTR4-F/FocTR4-R aforementioned primer set. The thermal cycling program consisted of the following steps: 94°C for 3 min, 35 cycles of 94°C for 30 s, 60°C for 30 s 72°C for 45 s, and a final extension at 72°C for 7 min. The PCR products were cloned into pMD18-T vector (Takara) according to the manufacturer’s instructions. The recombinant plasmid, designated pMD18-T-TR4, was used to make dilutions as a reference for evaluating the detection limits of the RealAmp assay and real-time PCR, respectively.

It is difficult to quantify Foc TR4 genomic DNA in soil samples, thus, the plasmid DNA mixed with soil DNA was used as both RealAmp and real-time PCR references to evaluate the sensitivity. While some inhibitory compounds exist in soil samples, mixing plasmid DNA with extracted soil DNA is a convenient approach to evaluate the detection limit of either RealAmp or real-time PCR. For the RealAmp assay, the pMD18-T-TR4 plasmid DNA was adjusted to the concentration of 430 ng/µl, and diluted into a 10-fold series (1×10^0^ to 1×10^7^ copies) before mixing with extracted soil DNA using a reference to assess the detection limit of the RealAmp assay, in comparison with real-time PCR. The standard curve was constructed according to the serial dilutions of extracted genomic DNA.

### Real-time PCR

The Real-time PCR assay was designed in this study with the SYBR® Premix *Ex Taq*™ kit (Takara, Dalian, China) and performed using the PRISM® 7500 Fast Real-Time PCR (Applied Biosystems) in a total volume of 25 µl following the manufacturer’s instructions. In this study, The Foc race 4 specific primer set FocSc-1/FocSc-2 (5′-CAGGGGATGTATGAGGAGGCTAGGCTA/5′-GTGACAGCGTCGTCTAGTTCCTTGGAG) described by Lin *et al.* (2013) was used, which was designed based on a SCAR marker sequence (named as Foc_242_) and have been confirmed to be highly specificity to Foc race 4 [43]. A 10-fold dilution series of plasmid DNA of Foc TR4 standard was analyzed in triplicate using the real-time PCR assay based on Ct values against the amount of plasmid DNA was then plotted to create a standard curve. A standard curve was constructed with eight ten-fold serial dilutions of the pMD18-T-TR4 plasmid DNA with soil DNA solution in triplicate real-time reactions described above. The thermal cycling conditions consisted of an initial denaturation for 5 min at 95°C, followed by 40 cycles at 95°C for 15 s annealing at 60°C for 30 s, extension at 72°C for 15 s. After the real-time PCR, melting curves (65°C to 99°C) of the PCR products were analyzed to verify their specificity.

### Feasibility Test

To investigate the availability of RealAmp assay for the detection of Foc TR4 in field surveys, a systematic survey for Foc TR4 was conducted in a total of 136 field samples in the banana-growing areas from South China in 2010-2011 (Table S1).The field-collected soil samples were quantitatively analyzed with both RealAmp assay and real-time PCR, respectively. All the data was analyzed using SPSS for windows 17.0 (SPSS Inc.). Independent *t*-test and paired *t*-test was used to test the significance between two methods (RealAmp and real-time PCR) at the P<0.05 level for each sample and all the samples, respectively.

## Results

### Development of RealAmp Assay for the Detection on Foc TR4

**Figure 2:**
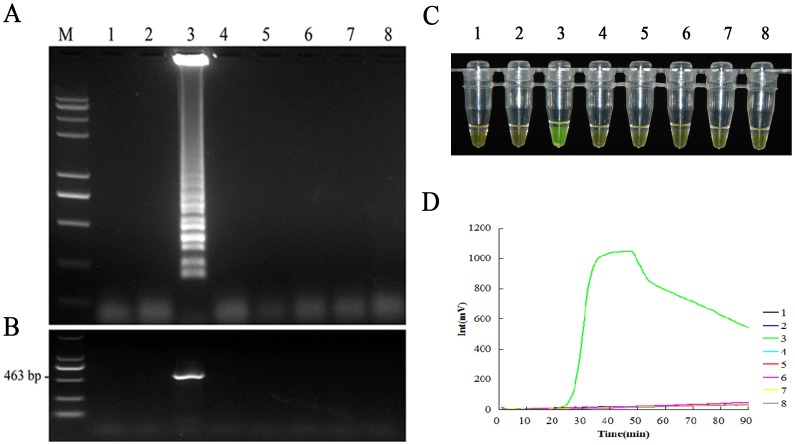
Specificity test of the real-time fluorescence loop mediated isothermal amplification assay (RealAmp assay) for the detection of Foc TR4. (A) Agarose gel electrophoresis analysis of RealAmp assay amplicon showing the specificity of RealAmp assay for detection of Foc TR4. Lanes 1–3, genomic DNAs of *Fusarium oxysporum* f. sp. *cubense* (Foc) race 1, subtropical race 4 (ST4) and tropical race 4 (TR4), respectively; Lanes 4–8, the DNAs of *Mycosphaerella melonis*, *Fusarium oxysporum* f. sp. *cucumerium, Fusarium oxysporum* f. sp. *luffae,* and *Fusarium oxysporum* f. sp. *niveum*, respectively; Lane M, Trans2K *plus* II DNA marker. Samples shown in lanes 1 to 8 in Fig. 2B, 2C and 2D is the same as in Fig. 2A. (B) The specificity of RealAmp assay was validated by specific PCR amplification using the specific FocTR4-F/FocTR4-R primer set. (C) Visual inspection of the RealAmp amplification products. The original orange colour of SYBR green turned green in the positive reaction mixture. (D) The fluorescence units vs. time graph plotted automatically by the ESE-Quant Tube Scanner. The graph reports the fluorescence in millivolts (mV) on the *y* axis and time in minutes on the *x* axis. Results can be read in the LCD panel as either positive or negative and/or in real time using a computer with the appropriate software.

The ESE-Tube scanner equipped with temperature settings to amplify DNA isothermally and spectral device to detect amplified product using fluorescence was applied for the detection of Foc TR4 in soil samples. In the specificity test, only amplified products from DNAs of Foc TR4 isolate were detected but not from DNAs of any tested isolates and other *formae speciales* of *F. oxysporum*, showing a high specificity of the designed primer set (Fig. 2A). The results were identical to those tested by specific PCR using the previously described specific primer pair. A single 463-bp product was amplified only from Foc TR4 isolates and no amplified bands were observed from other pathogens and water control (Fig. 2B). The colour of LAMP products of Foc TR4 isolate changed from orange to green when detected with SYBR Green I, whereas the colour of the other samples remained originally orange (Fig. 2C). The amplification curves obtained using the ESE-Quant tube scanner to monitor the DNA synthesis reaction indicated that the primer set were able to specifically amplify the target DNA sequence (Fig. 2D). Furthermore, the fragment from LAMP product was cloned into the pMD18-T vector and subsequently sequenced. The resulting sequence perfectly matched the sequence of IGS gene of Foc TR4 (data not shown), indicating that the RealAmp primer set was specific for Foc TR4, as no target products were amplified from the other DNAs of pathogens tested.

### Sensitivity and Standard Curve Analysis

**Figure 3:**
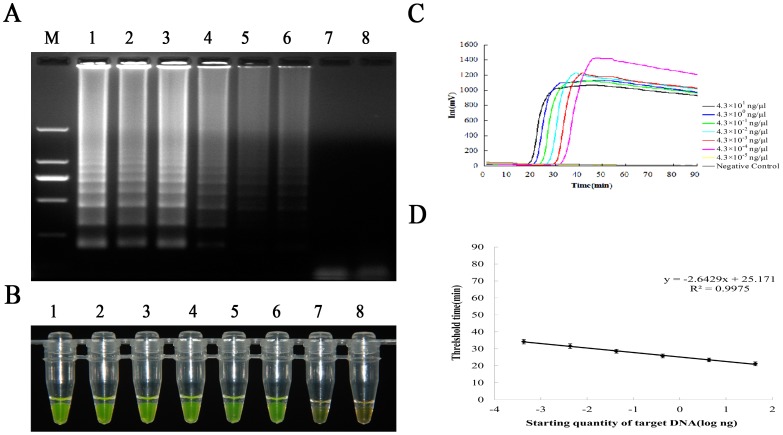
The sensitivity of RealAmp assay and standard curve. (A) Sensitivity test of RealAmp assay. Lane M, Trans2K *Plus* II DNA marker, lanes 1–8 correspond to serial 10-fold dilutions of Foc TR4 plasmid DNA ranging from 4.3 ng/µl to 4.3×10^−5^ ng/µl. Samples given in lanes 1 to 8 in Fig. 3B and 3C is the same as in Fig. 3A. (B) Visual detection of the RealAmp amplification products. The original orange colour of SYBR green turned green in the positive reaction mixture. (C) The fluorescence units vs. time amplification curves plotted automatically using an ESE-Quant Tube Scanner. (D) Standard curve for RealAmp assay. The threshold time (*T_t_*) vs. the amount of initial template plasmid DNA were plotted using an ESE-Quant Tuber Scanner. Error bars represent standard deviations from triplicate reactions.

For the sensitivity tests, serial dilutions of plasmid DNA mixed with extracted soil DNA were used to evaluate the sensitivity of the newly established RealAmp assay in comparison to the real-time PCR method. The electrophoresis showed that the RealAmp assay could detect as low as about 0.4 pg/µl of plasmid DNA when mixed with extracted soil DNA (Fig. 3A). The colour change of RealAmp products (from orange to green) was clearly observed in a range from 43 ng/µl of plasmid DNA to 4.3×10^−4 ^ng/µl plasmid DNA, which was identical to the electrophoresis result (Fig. 3B). This assay represented a standard fluorescence amplification of LAMP products with exponential growth using the ESE-Quant tube scanner (Fig. 3C). There was a good linearity between the threshold time (*T_t_*) and the initial amount of template plasmid DNA (R^2^>0.99, *P*<0.05), confirming that amplification was reliable and the RealAmp assay could be used for pathogen DNA quantification with traditional standard curves, and thus a standard curve between the threshold time (*T_t_*) and the amount of initial template plasmid DNA was constructed (Fig. 3D).

**Figure 4:**
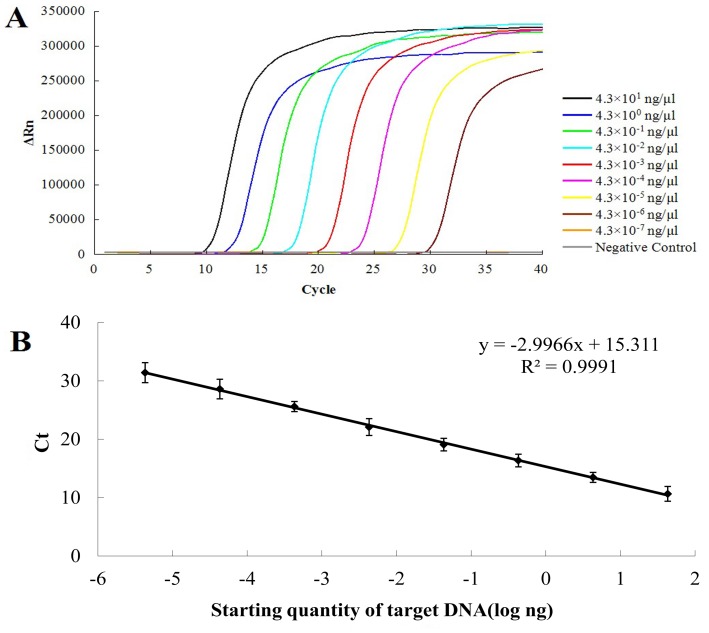
Determination of the detection limit of the real-time PCR and standard curve. (A) Sensitivity test of the real-time PCR. The real-time fluorescence units are plotted against concentration of initial plasmid DNA ranging from 4.3 ng/µl to 4.3×10^−6^ ng/µl using a PRISM® 7500 Fast real-time PCR. (B) Standard curve calculated from panel A. Standard curve generated using known concentration of 10-fold serially diluted pMD18-T-TR4 plasmid DNA and the threshold cycle (Ct) value. Every DNA concentration was measured 3-fold and error bars represent standard deviations from three replicate reactions.

Real-time PCR showed amplification of PCR products and regression analysis Ct versus initial template concentrations showed that the resulting standard curve was linear over a concentration range of at least 8 orders of magnitude, with the detection limit of the real-time PCR being approximately 4.3×10^−6^ ng/µl plasmid DNA when mixed with extracted soil DNA (Fig. 4A). The standard curve produced by the real-time PCR assay revealed a good linearity within the detection limit and a high correlation between Ct and DNA quantities (R^2^>0.99, *P*<0.05) (Fig. 4B). The detection limit of the real-time PCR was about 100-fold higher than that of the RealAmp assay. All the experiments were performed independently for three times (*n* = 3), and nearly identical results were obtained.

### Artificially Inoculated Soil Samples

**Figure 5:**
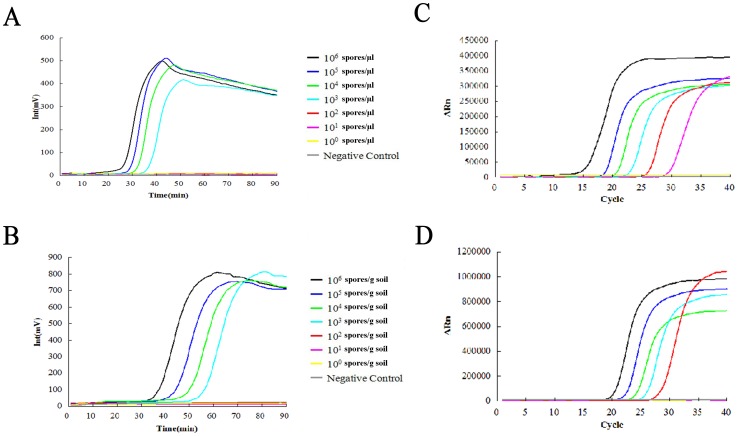
Detection of Foc TR4 from pure spores and spores in artificially infested soil using RealAmp assay and real-time PCR. (A) and (B) show detection of Foc TR4 from pure spores, respectively, using RealAmp assay. (C) and (D) show detection of Foc TR4 from artificially infested soil samples, respectively, using real-time PCR. (A) and (C) show the serial 10-fold dilutions of pure spores were ranging from 10^6^ to10^0^ spores/µl. (B) and (D) show the serial 10-fold dilutions of artificially infested soil samples were ranging from 10^5^ to10^0^ spores/g soil.

Artificially infected soil samples were prepared and the DNA was extracted as mentioned above. No Foc TR4 DNA was detected in the uninoculated control soil samples with either RealAmp assay or real-time PCR. The detection limit of RealAmp assay was 10^3^ spores/µl from pure spores and 10^3^ spores/g soil in artificially infested soil, indicating that strand displacement amplification conferred high tolerance and amplification efficiency for RealAmp assay (Fig. 5A, B). The detection limit of real-time PCR was 10 spores/µl from pure spores and 10^3^ spores/g soil in artificially infested soil (Fig. 5C, D).

### Detection in Field Soil Samples

To evaluate the effectiveness of the RealAmp assay for the detection of Foc TR4 in the agricultural soil samples, a total of 136 samples were tested by RealAmp and real-time PCR, respectively. 124 out of 136 samples were infected by Foc TR4. In the remaining 12 samples Foc TR4 was absent according to the RealAmp assay. Results from the real-time PCR assay indicated that 125 samples were positive and 11 samples were negative. The negative samples detected by real-time PCR were also tested negative by RealAmp assay. Only one sample was tested positive in real-time PCR were not detected by RealAmp assay. The detection rate of real-time PCR and RealAmp were 125/136 (91.9%) and 124/136 (91.2%) for the field samples in this study, respectively.

**Figure 6:**
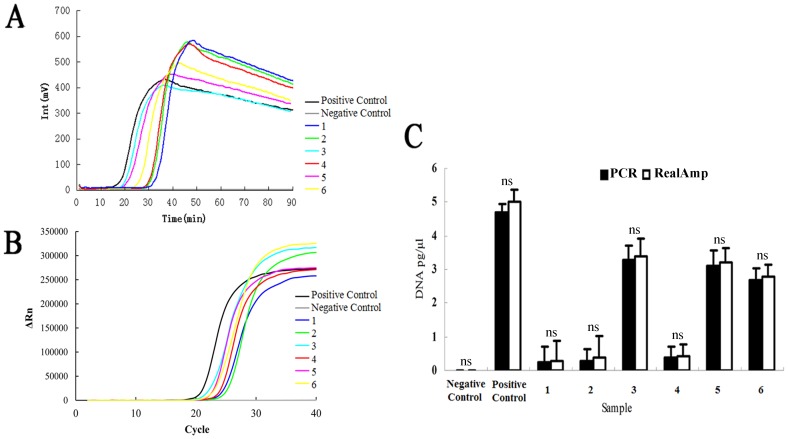
Detection of Foc TR4 in 6 randomly selected field soil samples using the RealAmp assay and real-time PCR method, respectively. (A) RealAmp assay detection of random field soil samples. The amplification curves were obtained using the ESE-Quant tube scanner. The graph reports the fluorescence in millivolts (mV) on the *y* axis and time in minutes on the *x* axis. Lanes 3 to 8 are random samples collected from different geographic locations, respectively. (B) Real-time PCR method detection of Foc TR4 in 6 randomly selected field soil samples and samples in lanes 3–8 correspond to those in panel (A). (C) Comparison of the quantitative results of 6 randomly selected samples between RealAmp assay and real-time PCR. A twice-autoclaved Foc-free soil sample served as negative control and a Foc TR4 artificially infested soil sample served as positive control in this study, respectively. Data are the mean (±SD) of three replicates. ns above columns indicate no significant difference between two detection methods for each sample at *P*<0.05 levels (ns, not significant). Paired t-test was used to test the significance of difference between two methods for all the soil samples at *P*<0.05 level.

The quantification of Foc TR4 DNA in soil between real-time PCR and RealAmp were statistically analyzed in 6 randomly selected samples with the SPSS software. No significant difference between the RealAmp and real-time PCR were observed based on quantitative results (Paried *t*-test, *P*>0.05) (Table S1, Fig. 6).

## Discussion

*Fusarium oxysporum* f. sp. *cubense* (Foc), especially tropical race 4 (TR4), is a ubiquitous soil borne pathogen which causes wilt disease on banana (*Musa* spp.) plants. Rapid and reliable detection of the pathogen is essential for undertaking appropriate and timely disease management measures. In recent years, various techniques have been developed to detect Foc-infected banana plant tissue, such as PCR-based methods [40]–[43] and LAMP assay [44]. However, no assay was developed to detect Foc race 4 in soil, which is a soil-borne pathogen that can survive for decades and cannot be controlled once a banana plant is infected.

In this study, a RealAmp assay was developed for the rapid and quantitative detection of Foc TR4 in soil. There is no requirement for require expensive reagents and equipment, compared with conventional real-time PCR. The ESE-Quant tube scanner provides a major advancement toward “electricity-free” technology for LAMP technology and offers a single-step amplification and product detection step. A portable fluorescent reader equipped with a battery pack (ESE-Quant Tube Scanner) is sufficient to run a RealAmp assay. The RealAmp assay we developed is the foundation of integrated disease management practice and can guide banana growers before planting and avoid further dissemination of Foc TR4.

The RealAmp assay is highly specific because it uses four primers that recognize six regions on the target DNA. The LAMP reaction is considered to progress through two steps by DNA polymerase with strand displacement activity: the starting structure producing step and the cycling amplification step. The outer primers, F3 and B3, recognize one of the six sites each and prime amplification of the entire region in a non-cycling manner. The inner primers, FIP and BIP, each recognize two of the six sites within the amplified sequence of the primer pair and form a dumbbell-like DNA structure used for subsequent cycling amplification. The LAMP prime set used in this study is a compromising consideration between detection specificity and amplification efficiency. On the one hand, the higher SNP frequency of the IGS region provides a rich source of genetic diversity in Foc, which was successfully exploited to develop a Foc TR4 specific PCR detection method by Dita *et al*. (2010) [42], and the designed FocTR4-F/FocTR4-R prime set used as outer prime in this study for the consideration of specificity. On the other hand, the outer prime set used in this study could amplify the Foc TR4 specific target IGS region and subsequently initiate the LAMP reaction, and the LAMP reaction processed at a constant temperature by one type of enzyme, the distance of outer primer from F2/B2 regions has no significant effect on amplification efficiency. Only amplified products from DNAs of Foc TR4 isolates showed ladder-like bands, while no amplicons were detected from DNAs of other tested *formae specials* of *F*.*oxysporum* and from the DNA-free control. Accordingly, the ESE-Quant Tube Scanner to monitor the DNA synthesis reaction using SYTO-9 fluorescence also indicated the primer set was specific to amplify the target DNA sequence. Additionally, the sequences of the smallest fragment amplified from field samples had a 100% sequence identity to the IGS region of Foc TR4 in GenBank (accession number FJ985561, data not shown). These results indicated this RealAmp assay was highly specific for diagnosis of Foc TR4.

Since *F. oxysporum* is a soil-borne pathogen, it is difficult to extract pure genomic DNA from spores to use as a standard. Therefore, the amount of pathogen DNA was quantified using a standard curve generated by including reactions containing different amounts of a plasmid carrying the Foc TR4 target sequence. While some inhibitory compounds exist in soil, mixing plasmid DNA with extracted soil DNA is a convenient approach to evaluate the detection limit of either RealAmp assay or real-time PCR. Thus, the plasmid DNA diluted with DNA extracted from twice-autoclaved soil was used as both RealAmp and real-time PCR references to evaluate the sensitivity of the Foc TR4. The RealAmp assay could detect as low as 0.4 pg/µl plasmid DNA mixed with soil DNA, which was 100 times lower than that of real-time PCR. Accordingly, the detection limit of real-time PCR was about 100-fold higher than that of RealAmp assay in pure spores. However, RealAmp assay with nearly same detection limit with real-time PCR for artificially infested soil, indicating the LAMP-based assay has an increased tolerance of inhibitory substances, compared with PCR-based methods [47].The increased tolerance to inhibitors also conferred a greater efficiency and convenience of the RealAmp assay for field surveys.

The RealAmp assay is a simple and accurate method for quantifying pathogen DNA in soil samples. Among 136 field-collected samples, quantitative results of 6 randomly selected field soil samples between RealAmp assay and real-time PCR method are shown in fig. 6 for demonstration of the usefulness of the newly established RealAmp assay (Fig. 6). Furthermore, the amount of DNA in a total of 136 soil samples were tested using both the RealAmp assay and real-time PCR method are shown in the Supplementary table 1, and no significant difference was found between two methods in quantifying the pathogen DNA in soil samples collected from the field (Paired test, *P*>0.05).

It is worth noting that two different intercalating fluorescent dyes in one assay in this study. In some studies, SYBR Green I was shown to have an inhibitory effect on enzymatic DNA amplification when used in a high concentration, and thus SYTO-9 fluorescent dye is a better choice than SYBR Green I for RealAmp assay [48]. However, the rapid and unambiguous visual inspection of LAMP results is essential for diagnostic and discrimination of positive samples in field. Then, an improved closed-tube visual inspection was achieved by addition of 1 µl SYBR Green I to the inside of the lid of the amplification tube prior to start of the reaction. After reaction, the SYBR Green I was added to LAMP reaction solution by gentle centrifugation at about 500 g for 10 s. Furthermore, the risk of cross-contamination is minimal using the improved closed-tube visual detection system, which facilitates rapid screening of samples without the use of gel electrophoresis or a fluorescence reader and would be helpful for high-throughput application. Moreover, the RealAmp assay had a high tolerance to inhibitors of DNA from soil samples. It would be a simple and effective approach for the quantitative detection and monitoring of Foc TR4 in soil avoiding further dissemination of Foc TR4 and would be useful for a routine soil-borne detection service in South China.

## Supporting Information

Table S1 **Quantification of 136 soil samples by RealAmp assay and real-time PCR method, respectively. Data are the mean (±SD) of three replicates.**
(XLS)
