# Correction: Development of a Real-Time Fluorescence Loop-Mediated Isothermal Amplification Assay for Rapid and Quantitative Detection of *Fusarium oxysporum* f. sp. *cubense* Tropical Race 4 In Soil

**DOI:** 10.1371/annotation/8a1fd6a3-754f-42e2-a906-9d224167344e

**Published:** 2014-01-02

**Authors:** Xin Zhang, He Zhang, Jinji Pu, Yanxiang Qi, Qunfang Yu, Yixian Xie, Jun Peng

Multiple funding organizations and grants were incorrectly omitted from the Funding Statement. The Funding Statement should read: "The work was supported by the Earmarked Fund for China Agriculture Research System (Project No. CARS-32-04), Keystone Science and Technology Plan of Hainan Province (ZDXM20130044), the National Natural Science Foundation of China (31301628) and the Central Nonprofit Scientific Research Institutes for Basic Scientific Research Special Fund (1630042013021). The funders had no role in study design, data collection and analysis, decision to publish, or preparation of the manuscript."

